# Head-to-head comparison of (*R*)-[^11^C]verapamil and [^18^F]MC225 in non-human primates, tracers for measuring P-glycoprotein function

**DOI:** 10.1007/s00259-021-05411-2

**Published:** 2021-06-11

**Authors:** Lara García-Varela, David Vállez García, Pablo Aguiar, Takeharu Kakiuchi, Hiroyuki Ohba, Norihiro Harada, Shingo Nishiyama, Tetsuro Tago, Philip H. Elsinga, Hideo Tsukada, Nicola A. Colabufo, Rudi A. J. O. Dierckx, Aren van Waarde, Jun Toyohara, Ronald Boellaard, Gert Luurtsema

**Affiliations:** 1 Department of Nuclear Medicine and Molecular Imaging, University of Groningen, University Medical Center Groningen, Hanzeplein 1, 9713 GZ Groningen, the Netherlands; 2 Department of Nuclear Medicine and Molecular Imaging Group, Clinical University Hospital, IDIS Health Research Institute, Santiago de Compostela, Spain; 3 Central Research Laboratory, Hamamatsu Photonics K. K, Hamamatsu, Japan; 4 Research Team for Neuroimaging, Tokyo Metropolitan Institute of Gerontology, Tokyo, Japan; 5 Dipartimento di Farmacia-Scienze del Farmaco, Università degli Studi di Bari, Bari, Italy

**Keywords:** Brain imaging, Central nervous system, Efflux transporter, Regional differences

## Abstract

**Purpose:**

P-glycoprotein (P-gp) function is altered in several brain disorders; thus, it is of interest to monitor the P-gp function in vivo using PET. (*R*)-[^11^C]verapamil is considered the gold standard tracer to measure the P-gp function; however, it presents some drawbacks that limit its use. New P-gp tracers have been developed with improved properties, such as [^18^F]MC225. This study compares the characteristics of (*R*)-[^11^C]verapamil and [^18^F]MC225 in the same subjects.

**Methods:**

Three non-human primates underwent 4 PET scans: 2 with (*R*)-[^11^C]verapamil and 2 with [^18^F]MC225, at baseline and after P-gp inhibition. The 30-min PET data were analyzed using 1-Tissue Compartment Model (1-TCM) and metabolite-corrected plasma as input function. Tracer kinetic parameters at baseline and after inhibition were compared. Regional differences and simplified methods to quantify the P-gp function were also assessed.

**Results:**

At baseline, [^18^F]MC225 V_T_ values were higher, and k_2_ values were lower than those of (*R*)-[^11^C]verapamil, whereas K_1_ values were not significantly different. After inhibition, V_T_ values of the 2 tracers were similar; however, (*R*)-[^11^C]verapamil K_1_ and k_2_ values were higher than those of [^18^F]MC225. Significant regional differences between tracers were found at baseline, which disappeared after inhibition. The positive slope of the SUV-TAC was positively correlated to the K_1_ and V_T_ of both tracers.

**Conclusion:**

[^18^F]MC225 and (*R*)-[^11^C]verapamil show comparable sensitivity to measure the P-gp function in non-human primates. Moreover, this study highlights the 30-min V_T_ as the best parameter to measure decreases in the P-gp function with both tracers. [^18^F]MC225 may become the first radiofluorinated tracer able to measure decreases and increases in the P-gp function due to its higher baseline V_T_.

**Supplementary Information:**

The online version contains supplementary material available at 10.1007/s00259-021-05411-2.

## Introduction

ATP binding cassette (ABC) transport proteins located at cerebral endothelial cells in the blood-brain barrier (BBB) pump out a wide variety of compounds from the brain to the blood [1, 2], contributing to the maintenance of cerebral homeostasis and the protection of the central nervous system (CNS) [3, 4]. These transporters use ATP hydrolysis to transport substrates from the intracellular to the extracellular compartment [5, 6]. P-glycoprotein (P-gp), breast cancer resistance protein (BCRP), and multidrug resistance-associated protein 1 (MRP-1) are the most studied ABC transporters, because of their clinical relevance [7, 8].

The P-gp transporter can recognize a large number of structurally diverse exogenous compounds such as anti-cancer, anti-epileptic, and antidepressant drugs and also endogenous compounds. Inflammatory responses, stress, therapeutic drugs, and diet can modify the expression and/or function of the P-gp transporter [9, 10]. An increase in the P-gp function has been related to decreases in drug efficiency (drug resistance) [1, 11]. This phenomenon is especially important in the treatment of brain tumors [12], intractable epilepsy [13], psychiatric diseases [1], and infectious diseases [14]. On the other hand, a decline in the P-gp activity is associated with an increased concentration of neurotoxic compounds inside the CNS, which may be related to the onset of several neurodegenerative diseases [15, 16] or may cause neurological problems [17, 18]. Vogelgesang et al. demonstrated that the β-amyloid deposition inside the brain, which is the pathological hallmark of Alzheimer’s disease [19, 20], is inversely correlated with the P-gp expression in endothelial cells of cerebral blood vessels [21].

Assessment of the P-gp function in vivo may help to diagnose several neurodegenerative diseases and may predict the efficacy of CNS treatments. PET imaging has already been used to study the P-gp function at the BBB in humans [22–26]. Nowadays, (*R*)-[^11^C]verapamil and [^11^C]-*N*-desmethyl-loperamide are considered “gold standard” tracers for imaging the P-gp function, being the most extensively used in preclinical and clinical research [24, 25, 27]. However, these tracers have been identified as strong P-gp substrates [22, 28, 29]; i.e., the tracers are quickly transported from the brain to the blood. This results in a low tracer concentration inside the brain [25], precluding their use in the assessment of P-gp upregulation, which might occur in treatment-resistant depression [30] and patients with intractable epilepsy [13].

For these reasons, many efforts have been made to develop new P-gp tracers with improved pharmacokinetic properties and lower affinity to the P-gp transporter [25]. [^11^C]metoclopramide [31], [^11^C]emopamil [32], [^11^C]phenytoin [26], and [^18^F]MC225 [33] were identified as weak substrates of the P-gp transporter, showing higher tracer uptake in the brain than (*R*)-[^11^C]verapamil at baseline conditions when P-gp is functioning adequately. [^18^F]MC225 was selected as the most promising fluorine-18 labeled tracer for in vivo measurement of P-gp function [34]. Recently, the kinetic properties of [^18^F]MC225 were evaluated, and the results confirmed the ability of this tracer to measure changes in the P-gp function of rats [33] and non-human primates [35].

The present study is the first direct comparison of the characteristics of the weak P-gp substrate [^18^F]MC225 and the strong P-gp substrate (*R*)-[^11^C]verapamil in non-human primates. To this aim, the function of the P-gp transporter was explored in three rhesus monkeys (*Macaca mulatta*) under normal conditions as well as after the administration of the P-gp inhibitor, tariquidar. Based on previous publications that analyzed the pharmacokinetics of [^18^F]MC225 and (*R*)-[^11^C]verapamil in non-human primates [35, 36], the 1-Tissue Compartment Model (1-TCM) was fitted to the data of both tracers. Kinetic parameters such as the influx constant K_1_, the volume of distribution (V_T_), and the efflux constant k_2_ were compared between the tracers at baseline and after inhibition. Regional differences between tracers were also analyzed, and simplified methods to quantify the P-gp function were assessed.

## Experimental section

### Tracer production

The radiosynthesis and quality control of (*R*)-[^11^C]verapamil and [^18^F]MC225 were performed as described previously [35–37].

### Animals experiments and study plan

All animal procedures were carried out in accordance with the recommendations of the National Institutes of Health (NIH), the guidelines of the Ethics Committee of the Central Research Laboratory, Hamamatsu Photonics (approval HPK-2016-07A), and the Institutional Animal Care and Use Committee of Tokyo Metropolitan Institute of Gerontology (approval 16,067).

**Fig. 1:**
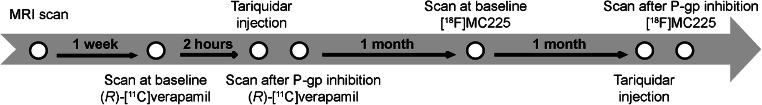
Schematic of PET protocol. First, each animal underwent an MRI scan (to acquire anatomic information), and subsequently, the PET scans were made, first using (R)-[^11^C]verapamil and later [^18^F]MC225

Three healthy male rhesus monkeys (*Macaca mulatta*; Hamri Co. Ltd., Ibaraki, Japan) were individually housed in a controlled room with a temperature of 24 ± 4 °C, a humidity of 50 ± 20%, and under a 14-h light/10-h dark cycle. Each monkey underwent a single MRI and 4 PET scans (Fig. 1) with (*R*)-[^11^C]verapamil and [^18^F]MC225. First, PET scans were performed using (*R*)-[^11^C]verapamil at baseline and after P-gp inhibition with tariquidar (after-inhibition scan). The interval between these scans was 2 h in two animals and 2 months in the third animal (due to technical problems). Approximately 1 month later, PET scans with [^18^F]MC225 were acquired, with an interval of 1 month between the baseline and after-inhibition scan. Inhibition of the P-gp function was achieved by intravenous administration of the P-gp inhibitor tariquidar (MedChemExpress, New Jersey, USA) at a dose of 8 mg/kg body weight, 15 min before the PET scan.

### Imaging experiments

First, a T1-weighted brain MRI scan of each subject was acquired (Signa Excite HDTx 3.0 T scanner, GE Healthcare). Afterward, animals underwent a 60-min transmission scan using a rotating ^68^Ge/^68^Ga rod source followed by a 91-min dynamic emission PET scan (SHR-38000, Hamamatsu Photonics) with arterial blood sampling. Animals were injected with (*R*)-[^11^C]verapamil (954.2 ± 45.6 MBq, with 99.3 ± 0.2% radiochemical purity and a molar activity (MA) higher than 20 GBq/μmol), or [^18^F]MC225 (684 ± 64 MBq, with a purity of 97.6 ± 1.0% and a MA higher than 36 GBq/μmol), as a single bolus (over 30 s) via the saphenous vein, at the start of the emission scan (for more information [35, 36]).

### Arterial blood sampling and analysis

Immediately after administration of the tracer and during the PET scan, blood samples (0.5 ml) were drawn from a cannula placed in the posterior tibial artery. Plasma and blood were separated according to the protocol described elsewhere [35]. Parent fraction and radioactive polar metabolites of (*R*)-[^11^C]verapamil and [^18^F]MC225 in plasma were determined as previously described [35, 36]. Standardized uptake values (SUV) time-activity curves (TAC) were calculated as previously described [35, 36].

### Image preprocessing

PET data were corrected for attenuation, using the transmission scan. Reconstruction of PET images, image registration, and analysis were performed as previously described [35, 36]. Briefly, MRI images were individually co-registered to an anatomical atlas and to the PET images. Several volumes of interest (VOIs) were extracted from the MRI data co-registered to the brain atlas [38].

### PET imaging analysis

#### Simplified quantification methods: standardized uptake values

For each PET scan, TACs were generated for the brain regions and corrected for the body weight and the dose of radioactivity injected to obtain SUV-TACs. Maximum values of each SUV-TAC of the 30 min scan were also extracted for both tracers from baseline and after-inhibition scans.

#### Pharmacokinetic modeling

The metabolite-corrected plasma and the whole-blood TACs were used as an input function to perform pharmacokinetic modeling using PMOD (PMOD Technologies, v3.8, Zürich, Switzerland).

Previous publications have already selected the 1-TCM to fit the (*R*)-[^11^C]verapamil and [^18^F]MC225 data of baseline and after-inhibition scans using short scan durations (<30 min) [35, 36]. Longer scan durations are more affected by the metabolism of the tracers since radio-metabolites can interfere with the brain signal and lead to erroneous measurements. Thus, this study focuses on the 30-min scan duration, and the 1-TCM was fitted to regional TACs of both tracers.

K_1_, V_T_, and k_2_ values from each brain region were compared between tracers at baseline and in after-inhibition scans. The effect size caused by P-gp inhibition was also compared between tracers. Moreover, a global scaling of the K_1_, V_T_, and k_2_ values was applied to study the regional differences relative to the whole-brain for both tracers and scans. To this aim, the K_1_, V_T_, and k_2_ values of each region were normalized to the values of the whole-brain (e.g., normalized K_1,i_ = VOI_i_ * K_1,i_/ VOI _wb_ * K_1,wb_ being VOI_i_ the volume of the region *i*, K_1,i_ the value of the region *i* VOI _wb_ the volume of the whole-brain and K_1,wb_ the value of the whole-brain). Relative regional differences between the tracers were compared in baseline and in after-inhibition scans. Regional changes caused by the P-gp inhibition were also related to the changes in the whole-brain and were compared between tracers.

The negative slope (also called the elimination constant (Ke_b_)) calculated using the SUV-TAC of the brain regions has been proposed as a simplified method to measure the P-gp function [39, 40]. We also calculated by linear regression the positive slope of regional SUV-TACs using the first 75 s of the scan. The slopes were compared to the K_1_ and V_T_ values of both tracers.

### Parametric images

An average parametric image representing K_1_, V_T_, and k_2_ values of the brain was calculated for both tracers and scans (baseline and after-inhibition) using 30-min scan duration. The metabolite-corrected plasma TAC was used as an input function for 1-TCM basis functions, using PMOD. Moreover, parametric images showing the changes in K_1_, V_T_, and k_2_ due to P-gp inhibition relative to baseline were also calculated using MATLAB (The MathWorks, Inc.). These images highlight the regions most affected by the P-gp inhibition for each tracer.

### Statistical analysis

Results are presented as mean ± standard error (SE) unless mentioned otherwise. Statistical analysis was performed using IBM SPSS Statistics version 23 (Armonk, NY, USA). Differences between tracers and scans were assessed for each brain region by generalized estimated equation (GEE) with independent matrix [41, 42]. Results were considered statistically significant at *p* < 0.05, without correction for multiple comparisons. The differences between baseline and after-inhibition scans were calculated as follows: *(V*_*T*_ *after-inhibition-V*_*T*_ *Baseline)/V*_*T*_ *Baseline*. The differences between tracers were calculated as *(V*_*T*_ *[*^*18*^*F]MC225-V*_*T*_ *(R)-[*^*11*^*C]verapamil)/V*_*T*_ *(R)-[*^*11*^*C]verapamil*, taking (*R*)-[^11^C]verapamil as the reference. Both values are expressed as percentages.

## Results

### Whole-brain SUV-TACs

**Fig. 2:**
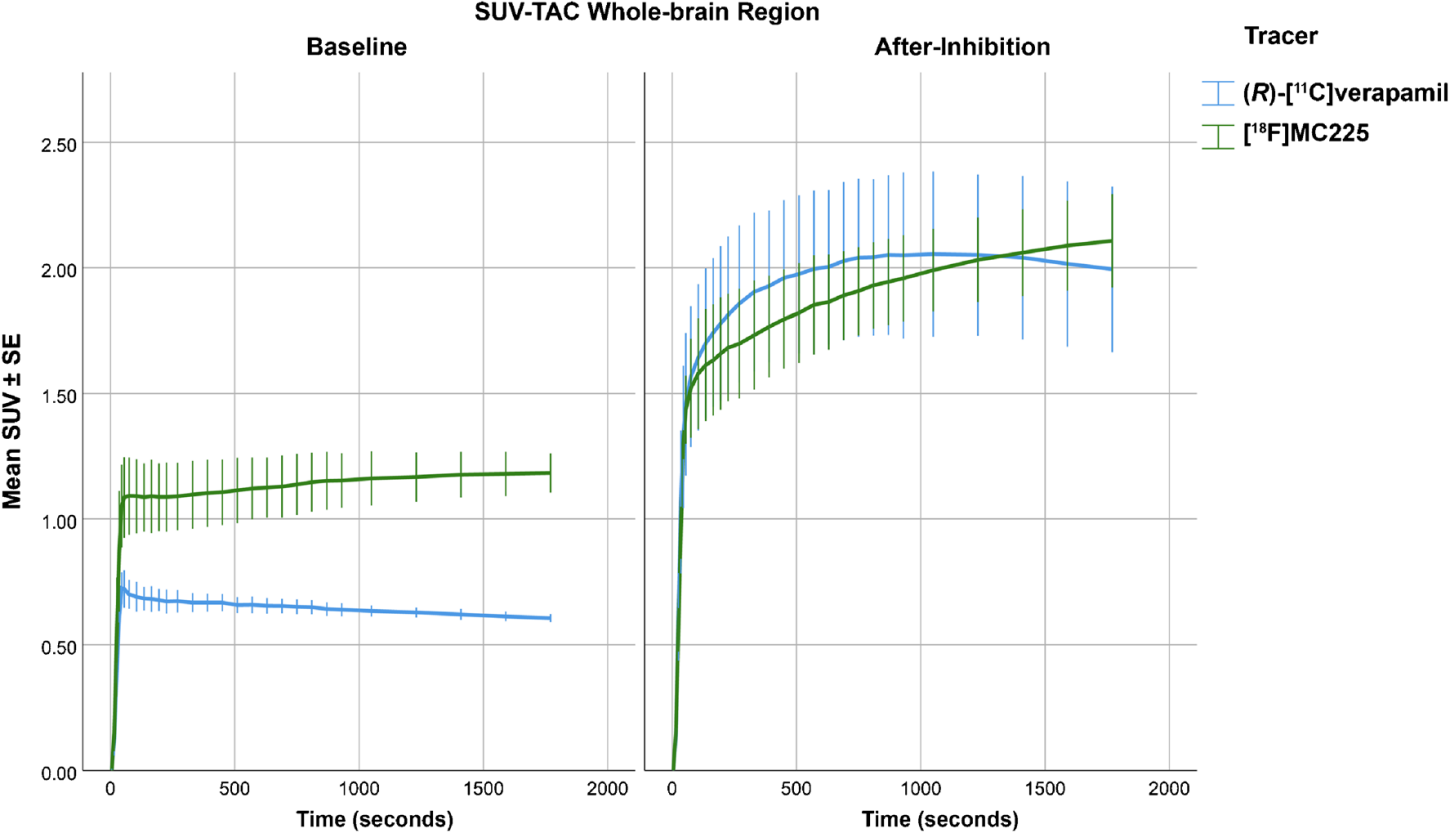
SUV-TAC of the whole-brain for both tracers at baseline (left) and after P-gp-inhibition (right)

At baseline, [^18^F]MC225 maximum SUV values were 64% (*p* < 0.001) higher than those of (*R*)-[^11^C]verapamil. [^18^F]MC225 SUV values continue to slightly increase after the initial sharp rise to a final value of 1.19 ± 0.03. In (*R*)-[^11^C]verapamil, the initial sharp rise led to a maximum value of 0.73 ± 0.03, which was reached at 48 ± 6 s. Tariquidar significantly increased the SUV-TACs for the whole-brain of both tracers (Fig. 2). The maximum values increased by 82% (SUV = 2.17 ± 0.10) for [^18^F]MC225 and by 184% (SUV = 2.07 ± 0.13) for (*R*)-[^11^C]verapamil. Moreover, the shape of the SUV-TACs completely changed after P-gp inhibition. In the case of [^18^F]MC225, a steeper increase was observed compared to baseline scans. In the case of (*R*)-[^11^C]verapamil, P-gp inhibition caused a delay in the peak value (from 48 ± 6 s to 17 ± 6 min). The maximum SUV values of the two tracers were not significantly different in after-inhibition scans (*p* = 0.155).

### Brain kinetics of [^18^F]MC225 and (*R*)-[^11^C]verapamil

#### Global changes in K_1_, k_2_, and V_T_

**Fig. 3:**
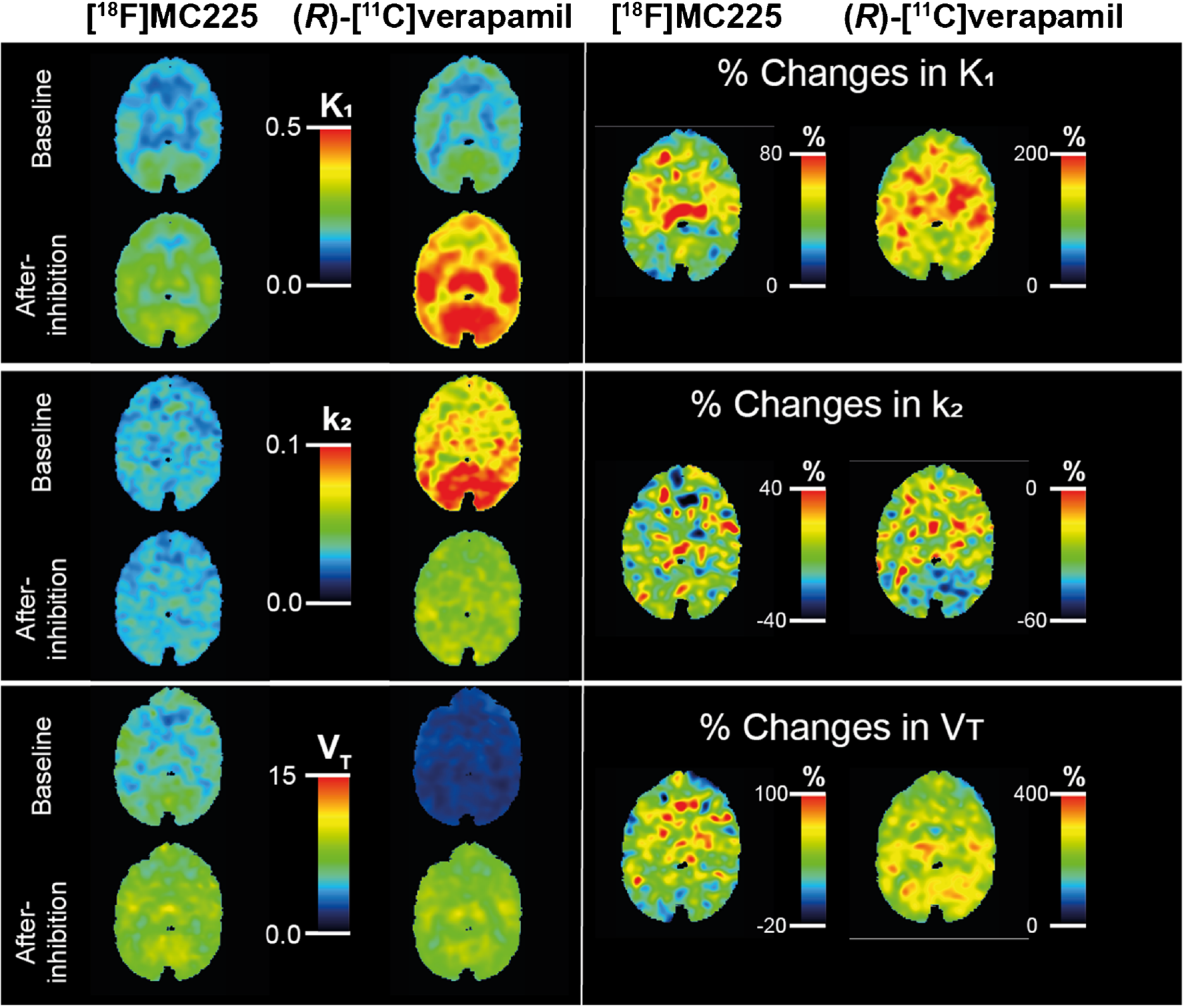
Parametric maps of K_1_ (above), k_2_ (middle), and V_T_ (below) of both tracers at baseline and after inhibition (left). Percentage of change due to the P-gp inhibition in K_1_ (above), k_2_ (middle), and V_T_ (below) for [^18^F]MC225 and (R)-[^11^C]verapamil (right)

Figure 3 shows the average parametric maps of K_1_, k_2_, and V_T_ of both tracers. The figure shows how the V_T_ and K_1_ values of both tracers increased after P-gp inhibition and (*R*)-[^11^C]verapamil k_2_ values decreased. At baseline, whole-brain V_T_ values were 140% higher for [^18^F]MC225 (6.05 ± 0.45) than for (*R*)-[^11^C]verapamil (2.52 ± 0.32) (*p* < 0.001), while k_2_ values were 59% lower for [^18^F]MC225 (0.03 ± 0.01) than for (*R*)-[^11^C]verapamil (0.07 ± 0.001) (*p* < 0.001). By contrast, whole-brain K_1_ values of both tracers at baseline were not significantly different (*p* = 0.718) (K_1_ [^18^F]MC225 = 0.17 ± 0.01 and K_1_ (*R*)-[^11^C]verapamil = 0.18 ± 0.03). After P-gp inhibition, the whole-brain K_1_ and the k_2_ of [^18^F]MC225 were 49% and 37% lower than those of (*R*)-[^11^C]verapamil (K_1_ [^18^F]MC225 = 0.24 ± 0.01 and K_1_ (*R*)-[^11^C]verapamil = 0.42 ± 0.05; *p* < 0.001) (k_2_ [^18^F]MC225 = 0.03 ± 0.001 and k_2_ (*R*)-[^11^C]verapamil = 0.05 ± 0.004; *p* < 0.001). Meanwhile, the whole-brain V_T_ of [^18^F]MC225 (8.04 ± 0.24) was not significantly different from the V_T_ of (*R*)-[^11^C]verapamil (8.87 ± 0.20) (*p* = 0.058).

Comparisons between V_T_, K_1_, and k_2_ values obtained from baseline and after-inhibition scans show that the effect size of the P-gp inhibition was larger in the case of (*R*)-[^11^C]verapamil. K_1_ increased up to 135.8% for (*R*)-[^11^C]verapamil (*p* < 0.001) after P-gp inhibition and up to 39.1% for [^18^F]MC225 (p < 0.001). V_T_ increased up to 252% for (*R*)-[^11^C]verapamil (p < 0.001) and to 33% for [^18^F]MC225 (*p* = 0.001). Values of k_2_ decreased by 32.9% for (*R*)-[^11^C]verapamil (p < 0.001) and did not significantly change for [^18^F]MC225 after P-gp inhibition. Supplemental Fig. 2 illustrates regional differences of K_1_, k_2_, and V_T_ for both tracers.

#### Regional changes in K_1_, k_2_, and V_T_

**Fig. 4:**
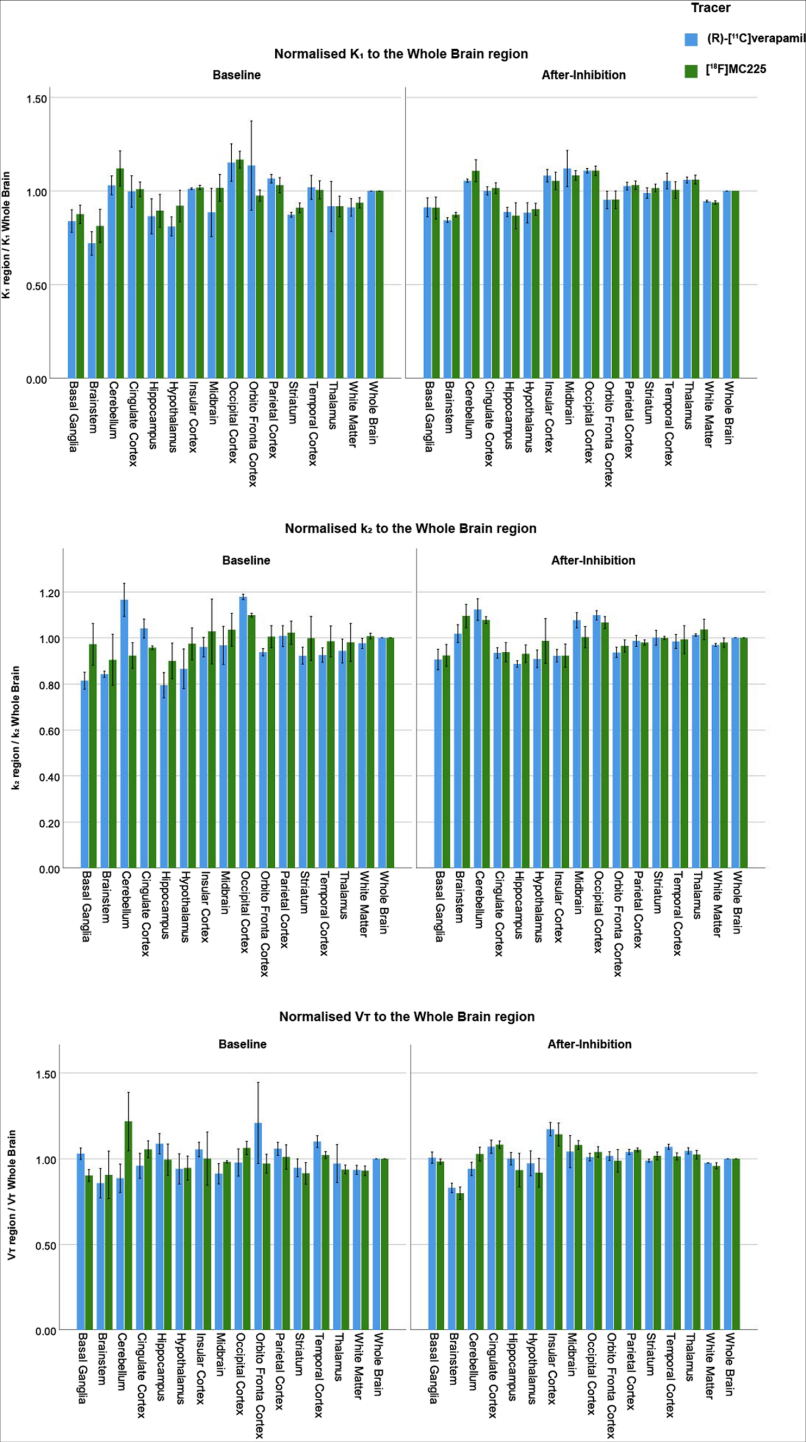
Regional differences in K_1_, k_2_, and V_T_ values relative to the whole-brain region at baseline and after inhibition with both tracers

As expected from Fig. 3, significant differences between brain regions were found for K_1_, k_2_, and V_T_ values. The spatial distribution of K_1_, k_2_, and V_T_ values and, in particular, the comparison between tracers and the impact of P-gp inhibition were carried out using the global changes as a confounder. Thus, K_1_, k_2_, and V_T_ values normalized to the whole-brain region of both tracers at both scans are represented in Fig. 4.

Regarding the baseline values of (*R*)-[^11^C]verapamil, regions such as the occipital and orbitofrontal cortex provided a higher relative contribution to the whole-brain K_1_, whereas, for the whole-brain V_T_, the largest contributions were from the orbitofrontal and temporal cortex. In the case of whole-brain k_2_ values, occipital cortex and cerebellum provided the highest relative contribution. Regarding the baseline K_1_ and V_T_ values of [^18^F]MC225, the regions with the highest contribution were occipital cortex and cerebellum, while occipital cortex and midbrain were the main contributors to the whole-brain baseline k_2_ values. These different relative contributions lead to regional differences between the tracers at baseline. For instance, in the case of [^18^F]MC225, the midbrain and orbitofrontal cortex K_1_ values were 15% higher and 14% lower than those of (*R*)-[^11^C]verapamil, respectively. Similarly, V_T_ values of [^18^F]MC225 were 38% higher in the cerebellum and 20% lower in the orbitofrontal cortex than those of (*R*)-[^11^C]verapamil. Also, k_2_ values of [^18^F]MC225 were 21% lower than those of (*R*)-[^11^C]verapamil in the cerebellum and 19% higher in the basal ganglia.

In after-inhibition scans, midbrain and occipital cortex provided the highest contribution to the whole-brain K_1_ of (*R*)-[^11^C]verapamil and occipital cortex and cerebellum for [^18^F]MC225. Insular and cingulate cortex were the regions with the highest contribution to the whole-brain V_T_ of both tracers. Regarding whole-brain k_2_ after-inhibition values, cerebellum and occipital cortex provided the highest contribution for (*R*)-[^11^C]verapamil, whereas brainstem and cerebellum provided the highest contribution for [^18^F]MC225. Although different relative contributions were also observed in after-inhibition scans, the regional differences between the tracers disappeared (regional differences between tracers were lower than 9%).

**Fig. 5:**
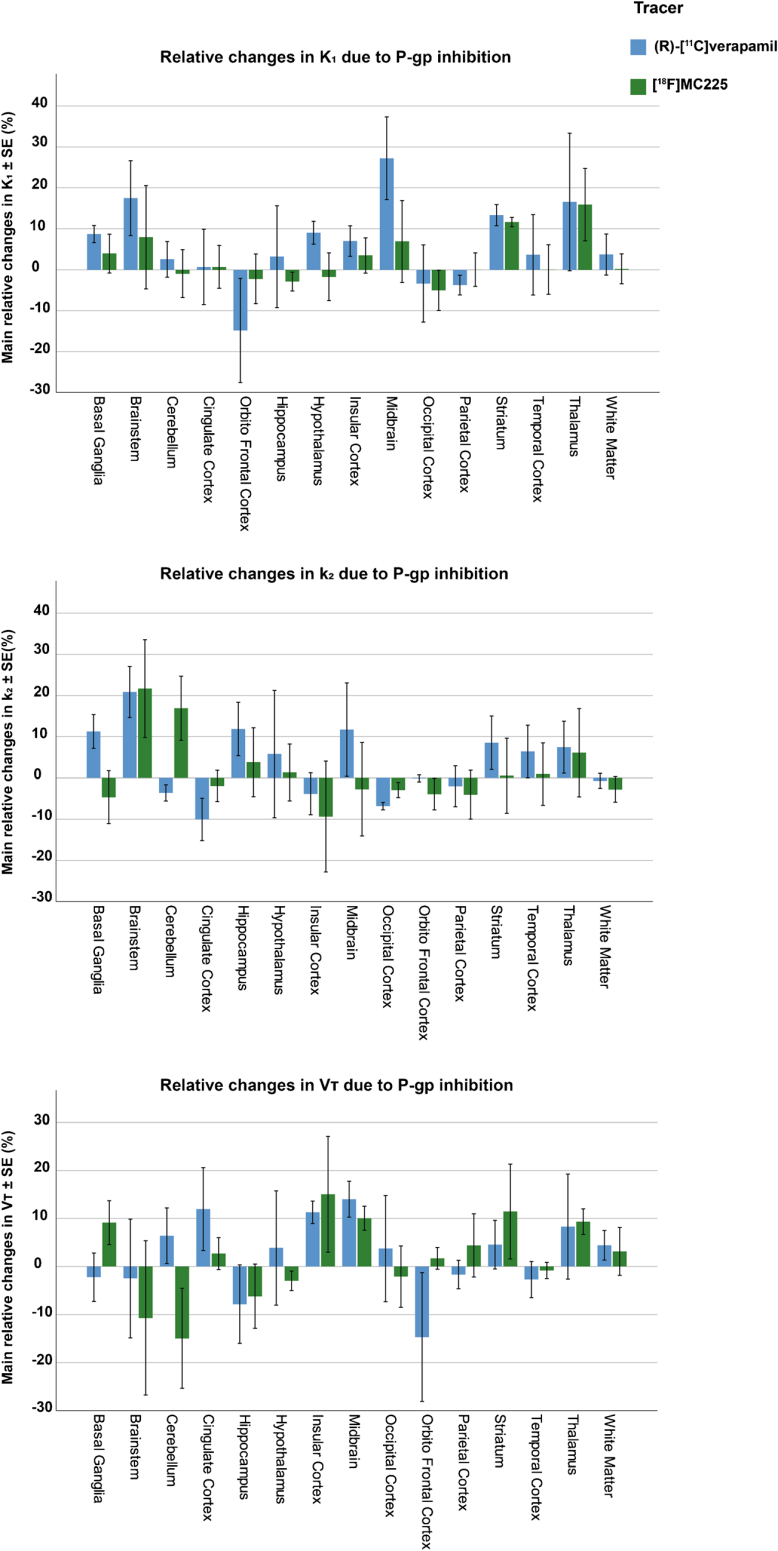
Relative changes of K_1_, k_2_, and V_T_ due to P-gp inhibition in all brain regions for the two tracers

Due to the differences in the [^18^F]MC225 K_1_, V_T_, and k_2_ values in various brain regions compared to the values of (*R*)-[^11^C]verapamil, the tracers showed also different changes of uptake due to the P-gp inhibition as can be observed in Fig. 5 and Supplemental Table 1.

### Correlations: SUV-kinetic parameters

Since the SUV-TAC did not show any negative slope during the 30 min of the scan duration, the Ke_b_ could not be calculated and correlated to the pharmacokinetic parameters. On the other hand, the positive slope of the SUV-TACs was positively correlated with the K_1_ (R^2^ (*R*)-[^11^C]verapamil = 0.63 (*p* < 0.001) and R^2^ [^18^F]MC225 = 0.61 (p < 0.001)) and V_T_ (R^2^ (*R*)-[^11^C]verapamil = 0.81 (p < 0.001) and R^2^ [^18^F]MC225 = 0.64 (p < 0.001)) values of both tracers (see Supplemental Fig. 3).

## Discussion

The present study compares the pharmacokinetic parameters of the novel P-gp PET tracer [^18^F]MC225 and the gold standard P-gp tracer (*R*)-[^11^C]verapamil in non-human primates, under normal conditions as well as after P-gp inhibition. The data of both tracers were assessed using a 30-min scan duration, to avoid the presence of radio-metabolites. The 1-TCM was the model fitted to the data of both tracers, following the recommendations in previous publications [35, 36]. The kinetic parameters (K_1_, k_2_, and V_T_) of the two tracers in baseline and after-inhibition scans were compared.

We found that in baseline scans, the V_T_ of [^18^F]MC225 was significantly higher than the V_T_ of (*R*)-[^11^C]verapamil. This finding was expected because (*R*)-[^11^C]verapamil is considered a strong substrate of P-gp and, consequently, is quickly transported from brain to blood, resulting in a low brain uptake. Meanwhile, [^18^F]MC225 is known as a weak substrate of P-gp transporter; thus, the baseline concentration of [^18^F]MC225 inside the brain is higher. This finding is in line with the previous studies performed in mice where the whole-brain SUV values of [^18^F]MC225 were higher than those of (*R*)-[^11^C]verapamil [34]. Nevertheless, our study showed that these differences in V_T_ do not arise from differences in K_1_, since K_1_ values were not significantly different between tracers at baseline. They are caused by differences in k_2_ values, which were significantly lower for [^18^F]MC225 than for (*R*)-[^11^C]verapamil.

Administration of the P-gp inhibitor tariquidar increased the V_T_ and K_1_ values of both tracers in all brain regions, while the efflux constant k_2_ was decreased for (*R*)-[^11^C]verapamil and remained unchanged for [^18^F]MC225. Since in 1-TCM, V_T_ = K_1_/k_2_, the rise in V_T_ after P-gp inhibition in [^18^F]MC225 scans is mainly caused by the increase in the K_1_. Based on the Fick principle and Renkin-Crone model [43, 44], K_1_ depends on the blood flow (F) and the extraction fraction (E_u_) of the tracer (K_1_ = F * E_u_). If K_1_ changes were caused by changes in the blood flow, then k_2_ values would also be increased (k_2_ = K_1_/V_T_). Since this was not the case, K_1_ changes seem to be caused by an increase in the extraction fraction of the tracer which can be related to increased permeability of the capillaries due to the P-gp inhibition. On the other hand, the increase in the (*R*)-[^11^C]verapamil V_T_ is caused by the increase in K_1_ and decrease in k_2_ values. The changes in k_2_ are negligible (−32%) compared to changes observed in V_T_ (+252%) and K_1_ (+136%). Thus, the increase in V_T_ is mainly related to the increase in K_1_ which is caused by the P-gp inhibition. For this reason, previous publications support the use of K_1_ as the best parameter to measure the P-gp function at the BBB, confirming the ability of both tracers to detect decreases in the P-gp function at the BBB of non-human primates [35, 36].

Even though both tracers can detect decreases in the P-gp function, the low V_T_ values of (*R*)-[^11^C]verapamil at baseline could hamper the quantification of increases in the P-gp function, which would be associated with a further decrease in tracer V_T_. [^18^F]MC225 does not show this limitation since it has a higher baseline V_T_ than (*R*)-[^11^C]verapamil. Moreover, the ability of [^18^F]MC225 to measure increases in the P-gp function has been confirmed, since the administration of a P-gp inducer to healthy rats decreased the V_T_ and K_1_ values of [^18^F]MC225 compared to controls [45]. Since K_1_ values at baseline were similar for both tracers, it could be expected that the (*R*)-[^11^C]verapamil K_1_ may be able to reflect increases in the P-gp function. However, most studies have failed to measure increases in the P-gp function using this parameter. For instance, (*R*)-[^11^C]verapamil scans in patients with chronic schizophrenia and major depression did not show significant differences in K_1_ values compared to controls [46, 47]. Also, (*R*)-[^11^C]verapamil was used to detect increased P-gp function in epileptic patients, but the authors did not find any significant differences in the K_1_ and V_T_ between epileptic brain tissue and its contralateral healthy tissue [48].

The present study also found that K_1_ and k_2_ values of (*R*)-[^11^C]verapamil were significantly higher than those of [^18^F]MC225, whereas V_T_ values of both tracers after P-gp inhibition were not significantly different. Since the animals were injected with the same dose of tariquidar (8 mg/Kg) and the same injection protocol was used, similar after-inhibition kinetic values were expected for both tracers, in particular, for K_1_ and V_T_. It was expected that after the P-gp inhibition, both tracers could enter the brain regions reaching similar values, as was the case for V_T_. Thus, one could argue that the K_1_ of (*R*)-[^11^C]verapamil may not be an adequate parameter to measure the P-gp function. The higher K_1_ values of (*R*)-[^11^C]verapamil after P-gp inhibition together with the lack of sensitivity of (*R*)-[^11^C]verapamil K_1_ values to detect increases in P-gp function may suggest that K_1_ of (*R*)-[^11^C]verapamil is affected by other non-specific factors. This conclusion is supported by in vitro studies which found that [^18^F]MC225 is more specific for P-gp than (*R*)-[^11^C]verapamil [34].

Our results also confirmed the presence of significant regional differences in the K_1_, V_T_, and k_2_ distributions for both tracers at baseline. Overall, the highest baseline V_T_ and K_1_ values for both tracers were found in cortical regions such as occipital and orbitofrontal cortex, and in the case of [^18^F]MC225, higher V_T_ and K_1_ values were also found in the cerebellum. These findings agree with previous publications where the highest uptake at baseline for both tracers in rats was found in frontal cortex and cerebellum [33, 49]. Since at baseline the P-gp function is working adequately, this may suggest that cerebellum and cortical regions display a lower P-gp function compared to other brain regions. However, after the P-gp inhibition, the regional differences between tracers were reduced, indicating that some regions were more affected by P-gp inhibition than others. In (*R*)-[^11^C]verapamil, the most affected region was the midbrain, whereas in [^18^F]MC225, it was the striatum. For both tracers, the regions less affected by P-gp inhibition were the cerebellum and orbitofrontal cortex. These results also suggest that subcortical areas may have a higher P-gp function than frontal cortex and cerebellum.

The study also described simplified quantification methods. SUV values do not reach a stable value during the 30-min PET scan, and therefore, they should not be used to estimate the P-gp function. Previous studies suggested the use of the negative slope of the whole-brain SUV-TAC as a simplified parameter to measure the P-gp function [39]. Since the 30-min SUV-TACs of both tracers did not show any washout, the negative slope could not be calculated. Instead, a positive slope using the first 75 s of the PET scan, which may be related to the entry of the tracer in the brain, was calculated and correlated to the kinetic parameters. The results showed a good correlation between the positive slope and the K_1_ and V_T_ of both tracers. Thus, the positive slope may be used as a surrogate parameter to estimate the P-gp function, avoiding the blood sampling and full kinetic analysis. This method may facilitate the assessment of the P-gp function in clinical studies.

Although both tracers were able to measure decreases in the P-gp function at the BBB of non-human primates, the use of fluorine-18 for the radiolabeling of [^18^F]MC225 may be advantageous. The longer half-life of fluorine-18 (T_1/2_ = 110 min) compared to carbon-11 (T_1/2_ = 20 min) allows the distribution of the tracer to remote PET centers and enables accurate plasma measurements. Furthermore, the lower maximum energy of the ^18^F isotope provides higher spatial resolution and thus higher quality of PET images [50, 51].

## Conclusion

This head-to-head comparison between [^18^F]MC225 and (*R*)-[^11^C]verapamil demonstrates that V_T_ calculated using 30-min scan duration may be a more adequate parameter than K_1_ to measure decreases in the P-gp function with both tracers. Although K_1_ was selected as the best parameter to measure the P-gp function in the previous publications [35, 36], the present study found that K_1_ was not different between tracers at baseline conditions, and therefore K_1_ could not be used to detect differences in affinity of strong and weak substrates towards the P-gp transporter. Moreover, K_1_ values of (*R*)-[^11^C]verapamil were higher than those from [^18^F]MC225 in after-inhibition scans, suggesting that (*R*)-[^11^C]verapamil K_1_ values may be affected by other non-specific unknown factors in the brain. The results from both tracers indicate that subcortical regions may present a higher P-gp function than frontal cortex and cerebellum. The higher baseline V_T_ of [^18^F]MC225 may allow the quantification of increases in the P-gp function and may facilitate the baseline image registration and fusion to an anatomical image (MRI or CT) (Supplemental Fig. 4). Thereby, [^18^F]MC225 has the potential to become the first radiofluorinated tracer able to measure both decreases and increases in P-gp function at the BBB. Nevertheless, a first-in-man study is required to verify the properties of [^18^F]MC225 before the tracer can be clinically applied.

## Supplementary information

ESM 1 (DOCX 1987 kb)

## Data Availability

The datasets used and/or analyzed during the current study are available from the corresponding author on reasonable request.
