# Supplementary material for: Head-to-head comparison of (R)-[11C]verapamil and [18F]MC225 in non-human primates, tracers for measuring P-glycoprotein function
Source: Eur J Nucl Med Mol Imaging. 2021 Jun 11;48(13):4307–17. doi: 10.1007/s00259-021-05411-2 (PMC8566421; doi:10.1007/s00259-021-05411-2)
Supplement: Supplementary file 1 — (DOCX 1987 kb) [file 259_2021_5411_MOESM1_ESM.docx]

**Supplemental material**

**Plasma Kinetics and Metabolism Analysis**

No significant differences between [^18^F]MC225 and (*R*)-[^11^C]verapamil were found in the SUV-TAC of whole-blood neither in baseline nor in after-inhibition scans. However, the metabolite-corrected SUV-TAC of plasma was higher for [^18^F]MC225 (SUVmean baseline: 2.88±0.28 and after-inhibition: 2.98±0.20) than for (*R*)-[^11^C]verapamil (SUVmean baseline: 2.00±0.35 and after-inhibition: 1.98±0.32) (p<0.001 in both cases).

No significant differences were found in the parent fraction TACs of both tracers at baseline or after-inhibition. At 30-min after tracer injection, the mean percentage of parent (*R*)-[^11^C]verapamil was (mean±SD) 56.8±6.9 % at baseline and 43.8 ±8.2 % after P-gp inhibition. A similar decrease of the parent fraction of [^18^F]MC225 was observed, from 53.2±2.2 % at baseline to 47.4±5.6% after P-gp inhibition. Supplemental Figure 1 shows the metabolite-corrected plasma SUV-TACs and parent fraction TACs of both tracers in baseline and after-inhibition scans.

The rate constant of elimination of the tracer from the blood (Ke) at baseline was higher for (*R*)-[^11^C]verapamil (Ke=0.0175) than for [^18^F]MC225 (Ke=0.0155) (p=0.005). After P-gp inhibition, the Ke values of both tracers were not significantly different (Ke (*R*)-[^11^C]verapamil = 0.0155 vs Ke [^18^F]MC225= 0.0147).


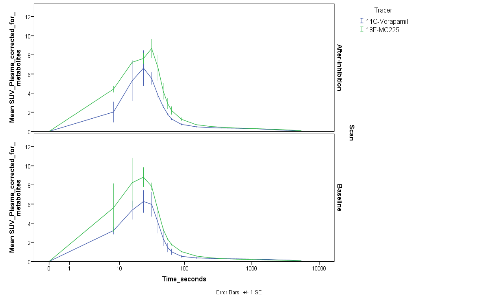

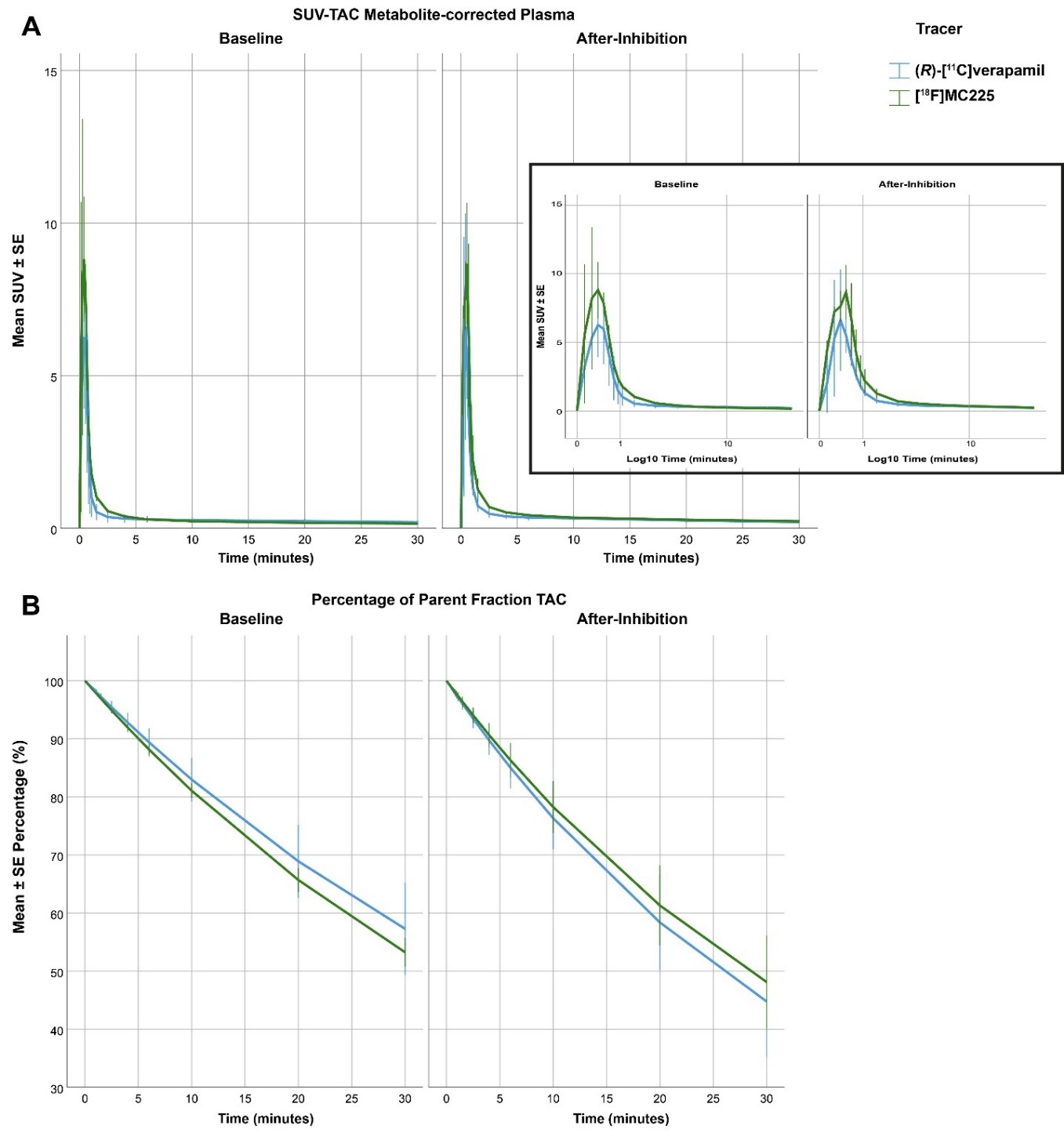


Supplemental Figure 1: Metabolite-corrected SUV-TACs of plasma for both tracers at baseline and after-inhibition (A). Parent fraction of [^18^F]MC225 and (R)-[^11^C]verapamil during the 30-min PET scan (B).


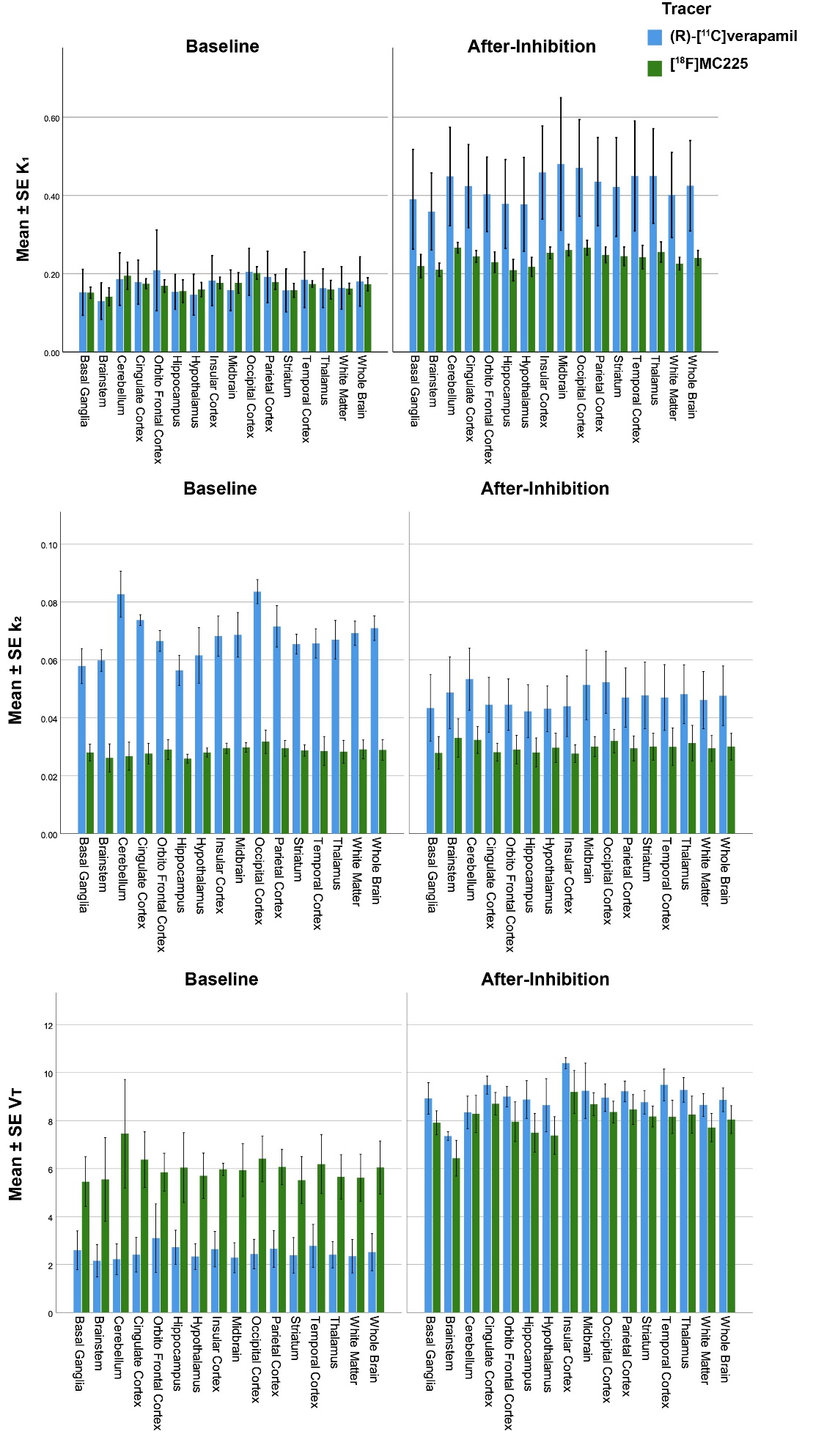


Supplemental Figure 2: K_1_ (A), V_T_ (B), and k_2_ (C) values of both tracers in different brain regions, at baseline (left) and after-inhibition (right).

Supplemental Table 1: Regional changes due to the P-gp inhibition in V_T_, k_2_, and K_1_ relative to the whole brain region in both tracers.

| **Relative Changes due to P-gp inhibition** | | | | | | |
| --- | --- | --- | --- | --- | --- | --- |
| **[^18^F]MC225** | | | | | | |
| **Region** | **Changes in K_1_** | **p-values** | **Changes in k_2_** | **p-values** | **Change V_T_** | **p-values** |
| **Basal Ganglia** | 4% | 0.035 | -5% | 0.069 | 9% | <0.001 |
| **Brainstem** | 7% | 0.129 | 21% | <0.001 | -12% | 0.079 |
| **Cerebellum** | -1% | 0.618 | 17% | <0.001 | -16% | 0.001 |
| **Cingulate Cortex** | 1% | 0.776 | -2% | 0.21 | 3% | 0.042 |
| **Hippocampus** | -3% | 0.004 | 3% | 0.287 | -6% | 0.019 |
| **Hypothalamus** | -2% | 0.427 | 1% | 0.648 | -3% | <0.001 |
| **Insular Cortex** | 3% | 0.048 | -10% | 0.072 | 14% | 0.001 |
| **Midbrain** | 7% | 0.085 | -3% | 0.498 | 10% | <0.001 |
| **Occipital Cortex** | -5% | 0.016 | -3% | <0.001 | -2% | 0.408 |
| **Orbito Frontal Cortex** | -2% | 0.35 | -4% | 0.015 | 2% | 0.06 |
| **Parietal Cortex** | 0% | 0.971 | -4% | 0.088 | 4% | 0.112 |
| **Striatum** | 12% | <0.001 | 0% | 0.979 | 11% | 0.002 |
| **Temporal Cortex** | 0% | 0.98 | 1% | 0.808 | -1% | 0.223 |
| **Thalamus** | 16% | <0.001 | 6% | 0.182 | 9% | <0.001 |
| **White Matter** | 0% | 0.917 | -3% | 0.026 | 3% | 0.119 |
| **Relative Changes due to P-gp inhibition** | | | | | | |
| **(R) -[^11^C]Verapamil** | | | | | | |
| **Region** | **Change K_1_** | **p-value** | **Change k_2_** | **p-value** | **Change V_T_** | **p-values** |
| **Basal Ganglia** | 9% | <0.001 | 11% | <0.001 | -2% | 0.268 |
| **Brainstem** | 17% | <0.001 | 21% | <0.001 | -3% | 0.558 |
| **Cerebellum** | 2% | 0.168 | -4% | <0.001 | 6% | 0.004 |
| **Cingulate Cortex** | 0% | 0.932 | -10% | <0.001 | 12% | <0.001 |
| **Hippocampus** | 3% | 0.6 | 12% | <0.001 | -8% | 0.025 |
| **Hypothalamus** | 9% | <0.001 | 5% | 0.385 | 3% | 0.487 |
| **Insular Cortex** | 7% | <0.001 | -4% | 0.06 | 11% | <0.001 |
| **Midbrain** | 27% | <0.001 | 11% | 0.006 | 14% | <0.001 |
| **Occipital Cortex** | -4% | 0.326 | -7% | <0.001 | 3% | 0.437 |
| **Orbito Frontal Cortex** | -16% | 0.021 | 0% | 0.765 | -16% | 0.026 |
| **Parietal Cortex** | -4% | <0.001 | -2% | 0.307 | -2% | 0.166 |
| **Striatum** | 13% | <0.001 | 8% | 0.001 | 4% | 0.029 |
| **Temporal Cortex** | 3% | 0.403 | 6% | 0.014 | -3% | 0.074 |
| **Thalamus** | 15% | 0.004 | 7% | 0.003 | 8% | 0.051 |
| **White Matter** | 4% | 0.062 | -1% | 0.325 | 4% | <0.001 |


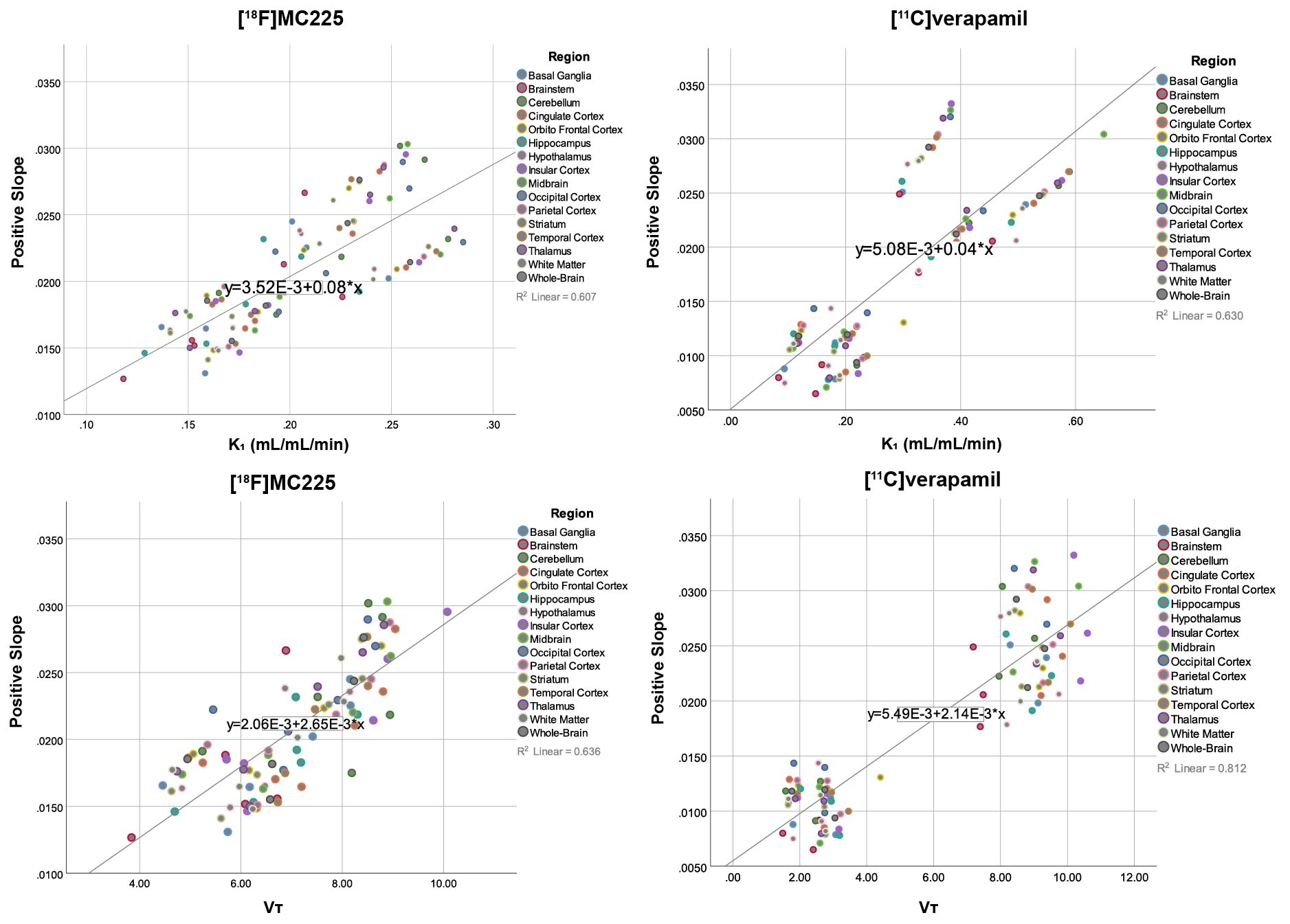


Supplemental Figure 3: Linear regression analysis of positive slope and the kinetic parameters obtained with 1-TCM.


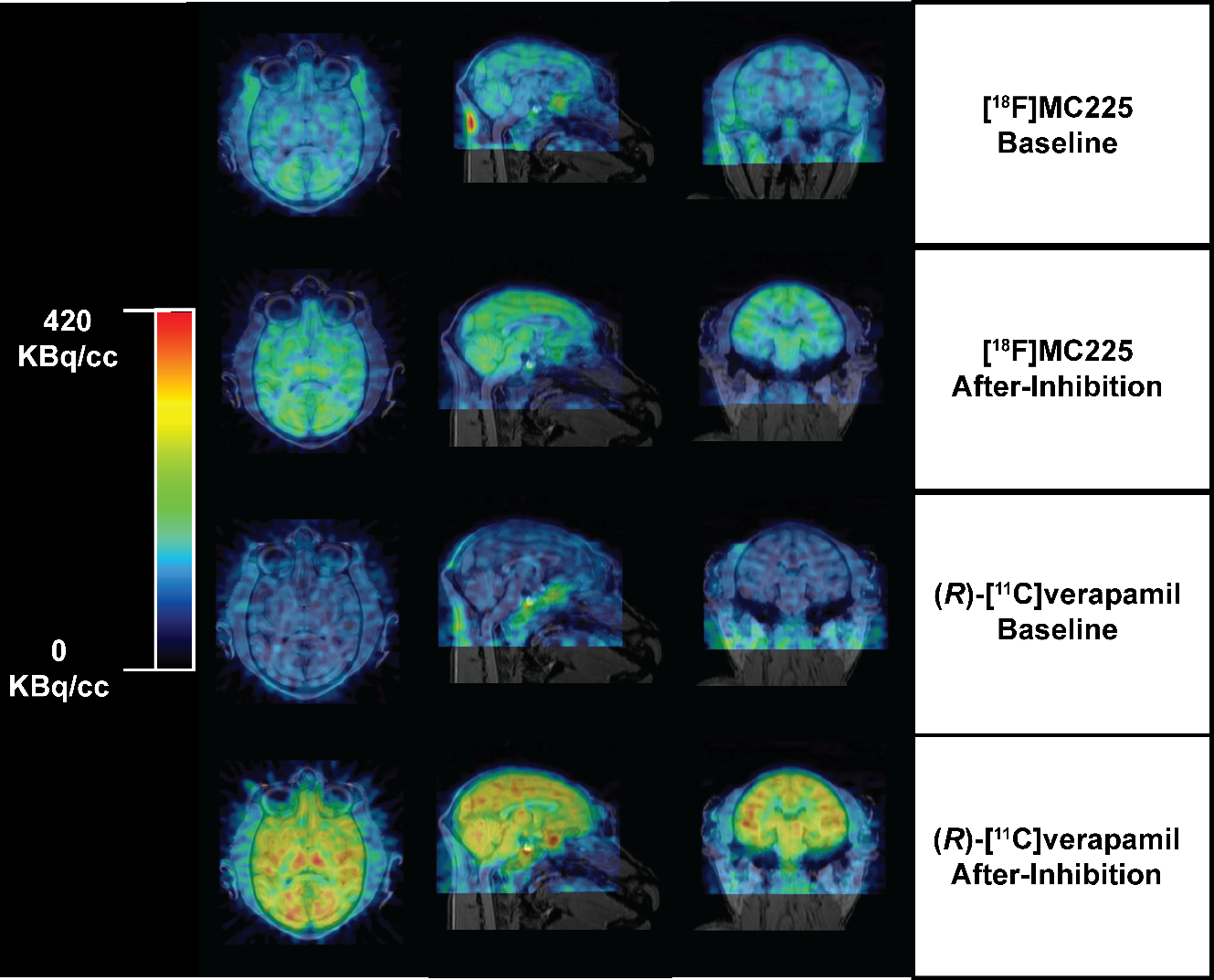


Supplemental Figure 4: Example of the fusion of the PET images of a subject to its corresponding MRI.
